# Pathophysiology and inflammatory biomarkers of sulfur mustard-induced corneal injury in rabbits

**DOI:** 10.1371/journal.pone.0258503

**Published:** 2021-10-12

**Authors:** Dinesh G. Goswami, Neha Mishra, Rama Kant, Chapla Agarwal, Claire R. Croutch, Robert W. Enzenauer, Mark J. Petrash, Neera Tewari-Singh, Rajesh Agarwal

**Affiliations:** 1 Department of Pharmaceutical Sciences, University of Colorado Anschutz Medical Campus, Aurora, Colorado, United States of America; 2 Medical Countermeasures Division, MRIGlobal, Kansas City, Missouri, United States of America; 3 Department of Ophthalmology, University of Colorado Anschutz Medical Campus, Aurora, Colorado, United States of America; Cedars-Sinai Medical Center, UNITED STATES

## Abstract

Sulfur mustard (SM) is a cytotoxic, vesicating, chemical warfare agent, first used in 1917; corneas are particularly vulnerable to SM exposure. They may develop inflammation, ulceration, neovascularization (NV), impaired vision, and partial/complete blindness depending upon the concentration of SM, exposure duration, and bio-physiological conditions of the eyes. Comprehensive *in vivo* studies have established ocular structural alterations, opacity, NV, and inflammation upon short durations (<4 min) of SM exposure. In this study, detailed analyses of histopathological alterations in corneal structure, keratocytes, inflammatory cells, blood vessels, and expressions of cyclooxygenase (COX)-2, matrix metalloproteinase (MMP)-9, vascular endothelial growth factor (VEGF), and cytokines were performed in New Zealand white rabbits, in a time-dependent manner till 28 days, post longer durations (5 and 7 min) of ocular SM exposure to establish quantifiable endpoints of injury and healing. Results indicated that SM exposure led to duration-dependent increases in corneal thickness, opacity, ulceration, epithelial-stromal separation, and epithelial degradation. Significant increases in NV, keratocyte death, blood vessels, and inflammatory markers (COX-2, MMP-9, VEGF, and interleukin-8) were also observed for both exposure durations compared to the controls. Collectively, these findings would benefit in temporal delineation of mechanisms underlying SM-induced corneal toxicity and provide models for testing therapeutic interventions.

## Introduction

Since its initial use as a chemical warfare agent (CWA) in the First World War, sulfur mustard (SM) has arguably been the most used CWA [1]. It has been coined, “the King of the Battle Gases” [2], because of its widespread use, like its deployment in the Berber rebellion in the 1920s [3], Italian- Ethiopian conflict in the 1930s [4], Egypt-Yemen conflict in the 1960s [5], Iran–Iraq war in the 1980s [6, 7], and Syrian conflict in 2000s [8, 9]. Exposure to this persistent, oily yellow-brown vesicant can occur through the eyes, skin, or via inhalation. SM (2,2′-dichloroethyl sulfide) is a bifunctional alkylating agent; once absorbed, it forms a charged, highly reactive intermediate, the ethylene episulfonium ion, that attacks and damages DNA, RNA, and proteins [1, 5, 10], causing cytotoxicity and inflammation, which may cause severe tissue damage [11–15].

Of the various routes of exposure to SM, ocular exposure is arguably the most damaging because of the aqueous nature of external ocular surfaces including the conjunctiva and cornea [16]; thus, making eyes plausibly the most susceptible organ to SM exposure. The preliminary toxic effects of SM on the eyes can manifest within minutes, depending upon the dose and duration of exposure and the ocular microenvironment [17]. The toxic effects can range from mild irritation, lacrimation, swelling/edema, inflammation, photophobia, blepharospasm, and corneal ulceration [18, 19] to delayed ocular effects, such as development of keratosis also known as mustard gas keratopathy (MGK), dryness, chronic inflammation, conjunctival scarring, epithelial injury, damage the corneal leading to its erosions, opacity, and neovascularization (NV), and limbal stem cell deficiency (LSCD) [20–25]. MGK has been observed in SM-exposed war veterans even 40 years after the initial exposure [21, 26].

Several studies to decipher the sequential progression of SM-related corneal injury and the underlying toxic mechanisms have been conducted. Hallmarks of SM-toxicity on rabbit eyes, such as changes in corneal thickness, corneal edema, corneal opacity, and corneal NV using different doses of SM for short durations (<4 min) have been well established [24, 25, 27–29]. However, studies characterizing the effects of SM exposure for longer durations were warranted; thus, in the present study we exposed the rabbit eyes for 5 min (shorter duration) and 7 min (longer duration) and performed comparative assessments of corneal thickness, corneal opacity, corneal ulceration, and corneal NV as well as epithelial degradation and epithelial-stromal separation, as a function of time for 28 days after SM exposure. Furthermore, we performed the histopathological analysis to examine the change in keratocyte numbers, inflammatory cell density, and blood vessel counts, along with the expression profiles of cyclooxygenase (COX)-2, matrix metalloproteinase (MMP)-9, vascular endothelial growth factor (VEGF). Although a few studies have assessed the expression levels of MMP-9 [24, 30, 31], VEGF [31, 32], and inflammatory responses [31, 33, 34], to our knowledge, this is the first study to analyze the expression levels of COX-2 and only the second study after Naderi *et al*. [35] to analyze keratocyte numbers in *in vivo* SM-exposed rabbit eyes. Notably, COX-2 is an enzyme that increases the production of prostaglandins during inflammation [36]; VEGF, as the name suggests, is extensively involved in angiogenesis and plays an important role in wound healing [37]; and MMP-9 belongs to the gelatinase group of metalloproteinases that are associated with disrupting the functioning of the corneal epithelial barrier, playing an important role in wound healing and inflammation [38].

Earlier, we have performed studies using nitrogen mustard (NM; bis-[2-chloroethyl] methylamine), a less toxic analogue of SM, on human corneal epithelial (HCE) cells [12], *ex vivo* organ culture model of rabbit cornea [39], and *in vivo* rabbit corneal injury model [40]. The *ex vivo* and *in vivo* corneal injury models with NM-exposure are integral in evaluating acute corneal injury and screening therapeutic modalities to alleviate and revert vesicant-induced ocular injury. However, it is critical to translate the toxic corneal effects from the NM model to the *in vivo* SM corneal injury model to transform the therapies identified in the NM model into the SM-induced injury model. Therefore, to understand the toxic effects as well as pathophysiology due to mild to moderate-severe SM exposure-durations, in the present study, we have developed *in vivo* rabbit cornea injury models using two SM vapor exposure durations; thus, employing a more consistent and efficient exposure system. Our studies are anticipated to help in further understanding the pathways involved in SM-induced acute and chronic corneal injuries, and in identifying and optimizing effective treatment options.

## Materials and methods

### SM exposure and clinical assessments

New Zealand white rabbits, weighing between 2.5–4.0 kg and a minimum of 3 months old, were obtained from Charles River Laboratories (Wilmington, MA). All the experimental protocols used in this study were approved by MRIGlobal’s IACUC. Following the pain management and anesthesia procedures approved by the MRIGlobal IACUC, all SM vapor exposures were performed under the chemical hood, based on MRIGlobal’s approved exposure protocol. Approximately 1 h before the commencement of SM exposure, buprenorphine-SR (SQ) was administered to the animals and every 72–96 h thereafter, as required. The animals were anesthetized at MRIGlobal’s chemical surety facility, using IM administration of ketamine+xylazine+acepromazine. Once anesthetized, the rabbits were positioned on an absorptive pad, and exposed to SM vapors by securing the ocular vapor goggles around their head, either for 5 or 7 min, inside the chemical hood. The left eye of the animal was treated as control (naïve or untreated) and the right eye was exposed to 400 μg/L SM (~390–420 μg/L; n = 6-7/per treatment group at each time point) for 5 min (short-duration exposure) or 7 min (long-duration exposure).

After the SM exposures, the rabbits were monitored daily and clinical observations were made, including assessments for signs of pain or discomfort. Clinical evaluations of injury to the whole eye and corneal were made; digital pictures were taken prior to SM exposure and thereafter at days 0 (6 h), 1, 3, 7, 14, 21, and 28 post both durations of SM-exposure. Parameters assessed to determine corneal injury included corneal ulceration, opacity, and NV as reported earlier [41]. Pachymetry measurements (TOMEY SP-3000 Pachymeter, Phoenix, AZ) were performed to measure the thickness of the unexposed and SM-exposed corneas, and assess any associated changes. Post SM exposure, rabbits were euthanized using pentobarbital overdose (using 1 ml of pentobarbital/Fatal Plus solution [IV] after anesthesia using either ketamine [25–35 mg/kg]/xylazine [5 mg/kg] or isofuorane [5%]), at day 1, 3, 7, 14, and 28, and corneas were removed and stored either by snap freezing in liquid nitrogen or by formalin fixation for further analysis.

### Histopathological evaluations

Histopathological evaluations of the corneal tissues were carried out after fixation and paraffin embedding as described previously [42]. Briefly, paraffin-embedded corneas were sectioned (5 μm thick), hematoxylin and eosin (H&E)-stained, and microscopic evaluations of thickness of cornea and epithelium along with degradation of the epithelium were performed. Corneal thickness was measured from 10–12 regions selected at random, at 100x magnification (~1.0 mm away from the limbus region). Epithelial thickness was determined using five regions (randomly chosen) from the entire corneal length (at 400x magnification). The value of epithelial thickness was determined by taking the average of five separate measurements from each of the five selected areas. A length of approximately 7 mm of the total corneal length was used to determine the epithelial degradation and epithelial-stromal separation.

### Estimation of keratocyte numbers, inflammatory cell density, and blood vessel count in the stroma

The number of keratocyte cells, inflammatory cells, and blood vessels was estimated from the stroma of the H&E-stained rabbit eye corneal sections (at 400x magnification). Keratocyte quantification was performed in ~7 mm^2^ area of the cornea; the numbers of blood vessels and inflammatory cells were estimated in the entire stromal region. The scoring of inflammatory cell density was 1: <50, 2: 50–100, 3: 100–500, 4: >500 inflammatory cells.

### Immunohistochemistry for COX-2, VEGF, and MMP-9

IHC for COX-2, VEGF, and MMP-9 was performed according to the protocol described previously [40] from the lab. Briefly, the corneal sections were first rehydrated, then heat-mediated epitope retrieval was performed, endogenous peroxidase activity was blocked, and sections were incubated with the respective primary antibodies [anti-VEGF (Cat#ab28775), anti-MMP-9 (Cat#ab58803) both from Abcam, Cambridge, MA, and anti-COX-2 (cat#160112; Cayman chemicals, Ann Arbor, MI); Rabbit IgG antibody (N-Universal, DAKO), negative control]. Thereafter, the sections were incubated sequentially using the streptavidin-biotin complex (ABC) IHC staining methodology for signal amplification. DAB was used for visualization of IHC (brown, cytoplasmic staining) followed by hematoxylin staining for the nucleus. The scoring for the cytoplasmic staining was performed in 10 randomly selected areas (400x magnification). This intensity of IHC staining was scored from 0 to 4; where a score of 0 signified no DAB staining (absence of any brown color) and 4 signified the maximum intensity of staining, described previously [40].

### Cell lysate preparation and measurement of cytokines

Cytokine arrays were performed from corneal tissues (n = 4–5) 3 days after SM exposure for 7 min. Lysates were prepared from the SM-exposed tissues and their respective controls; protein estimation was performed using the BioRad DC protein assay kit, following the manual instructions. The lysates were then quantified for cytokine levels using cytokine array kits (RayBiotech, Norcross, GA) according to the recommended protocol. Briefly, diluent was used to block the array epitopes. After blocking, the epitopes were incubated with the lysates. The signal detection was performed using a followed biotinylated antibody and streptavidin conjugated to a Cy3 equivalent dye. Thereafter, slides were scanned, and the data extraction was performed using RayBiotech scanning and data extraction services. RayBiotech array specific data analysis software was used for analysis and results were expressed as relative fluorescence units.

### Statistical analysis

One-way analysis of variance (ANOVA), with Tukey or Bonferroni T-test for multiple comparisons (SigmaStat 2.03) were performed to determine the statistical significance of the difference between the untreated control versus SM-exposed groups. Differences were considered statistically significant if the p<0.05. Data are represented as the mean±standard error of mean (SEM).

## Results

### SM exposure causes an increase in corneal thickness, corneal opacity, corneal ulceration, and corneal neovascularization

**Fig 1:**
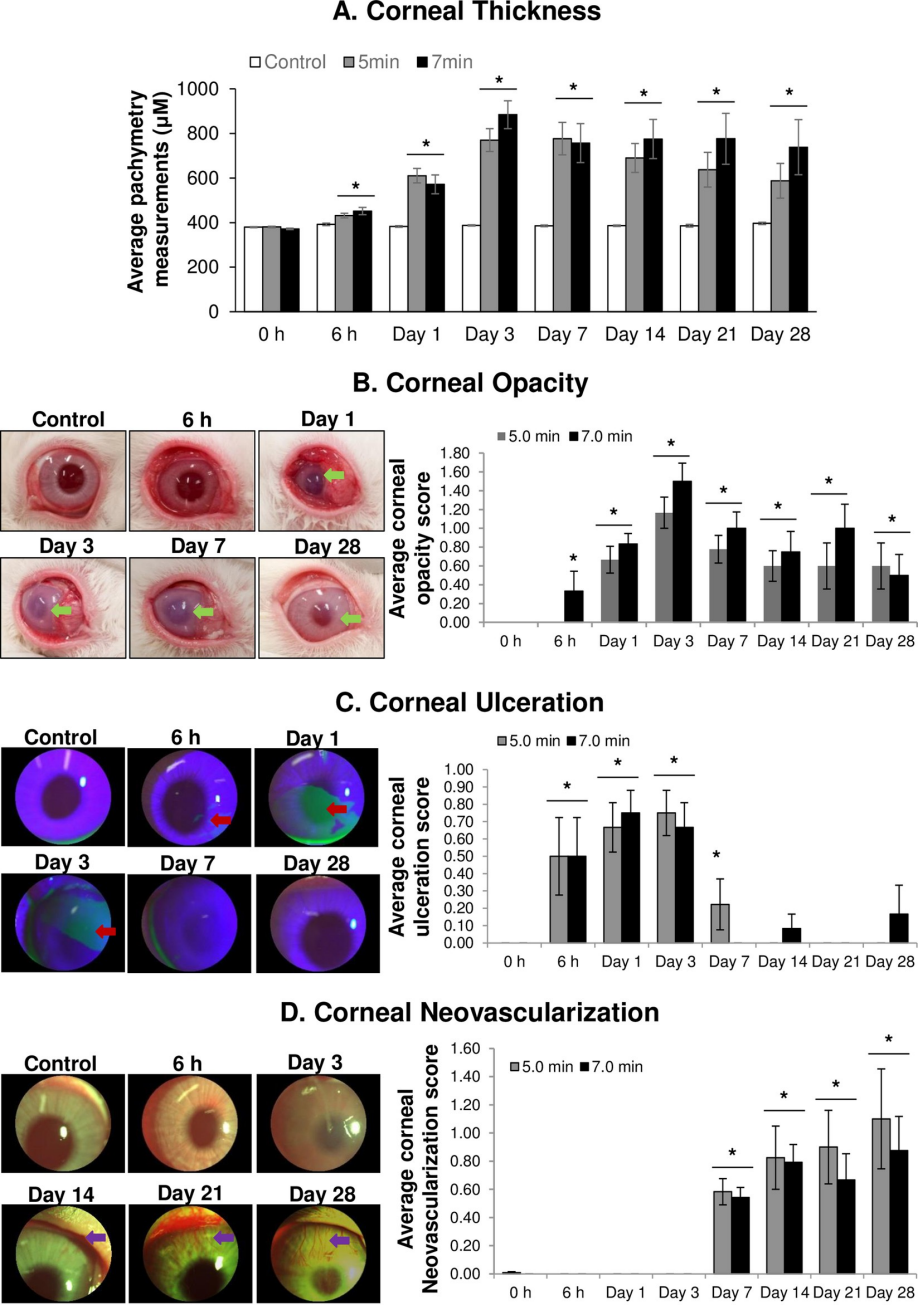
Ocular Sulfur Mustard (SM) exposure causes increase in corneal thickness and causes corneal opacity, corneal ulceration, and corneal neovascularization. The right eyes of New Zealand white rabbits were exposed to SM (~0.4 mg/L) vapor either for 5 min (shorter duration) or 7 min (longer duration) and the clinical progression of ocular injury was observed starting from day 1 (6 h) up to 28 days post-exposure, as detailed in the materials and methods section. The left eye was designated as the control eye and was exposed only to the dilution air. Corneal thickness (A), corneal opacity and quantification (B), corneal ulceration and quantification (C), and corneal neovascularization and quantification (D). Representative pictures are from the 7 min SM exposure group. Green arrows, corneal-stromal injury (opacity); red arrows, corneal ulceration; and purple arrows, corneal neovascularization. Data presented are mean±SEM (n = 5-7/group). *p<0.05 for both 5 min and 7 min exposure.

The effects of *in vivo* short i.e., 5 min and long i.e., 7 min durations of SM exposure on clinical measurements in rabbit cornea, as observed on 6 h to day 28 post SM exposure, are shown in Fig 1.

Pachymetry measurements showed that there was an overall increase in the corneal thickness upon both 5 and 7 min of SM exposure (Fig 1A and S1 Dataset). A slight increase was observed on 6 h and a significant increase was observed on day 1 post SM exposure for both the exposure durations as compared to their respective controls. The average corneal thicknesses on day 1 post-exposure were 611 μm and 572 μm for the 5- and 7-min exposure groups, respectively, compared to 383 μm for the controls. The corneal thickness remained significantly elevated throughout the study duration of 28 days post SM exposure for both the exposure durations (Fig 1A). From day 3 onwards, a slight dose-dependent effect was observed with maximal increase on day 3 for 7 min exposure duration (average corneal thicknesses were 877 μm and 387 μm for the SM exposure and control groups, respectively). For the 5 min exposure group, the maximal increase was on day 7 post SM exposure with the average corneal thickness of 776 μm for the SM exposure group compared to 385 μm average corneal thickness for the control group (Fig 1A). After day 7 in the 5 min SM exposure group, the corneal thickness gradually decreased although the thickness was still significantly more in the SM exposure group than the control group till the study end point of day 28 post-exposure (average corneal thicknesses were 690 μm, 638 μm, and 588 μm; respectively, on day 14, 21, and 28 post-exposure). In the 7 min exposure group, on day 7 post-exposure, a decrease in corneal thickness was observed (average corneal thickness: 757 μm). On day 14, the corneal thickness was elevated slightly (average corneal thickness: 775 μm) and remained sustained until day 21 post-exposure (average corneal thickness: 776 μm), before decreasing slightly on day 28 post-exposure (average corneal thickness: 738 μm; Fig 1A).

Corneal opacity (reduced transparency/increased clouding of the cornea due to corneal scarring) was evaluated post SM exposure in the rabbit cornea according to the scoring described previously [41]. Time-dependent effect was observed in corneal opacity following SM exposure (Fig 1B and S2 Dataset). Corneal opacity started to appear at 6 h post-exposure in 7 min exposure group, which reached its maximal value on day 3 post-exposure (average corneal opacity score = 1.5) and remained slightly higher than the 5 min exposure group at all the study time points except on day 28 post-exposure. In the 5 min exposure group, corneal opacity began to appear on day 1 post-exposure, and reached its maximal value on day 3 post-exposure (average corneal opacity score = 1.17), similar to that of the 7 min exposure group. The corneal opacity started to resolve after day 3 for both the exposure groups (Fig 1B). However, it did not fully resolve even at the study duration of day 28 post SM exposure in both the exposure groups.

Corneal ulceration was evaluated post SM exposure using slit lamp imaging (disruption of epithelial layer facilitates the stromal fluorescein staining) of eyes as described previously [41]. Significant corneal ulceration was observed at the earliest time point (6 h) post SM exposure for both the exposure durations (Fig 1C and S3 Dataset). Corneal ulceration peaked (average corneal ulceration score = 0.75) on days 1 and 3 post SM exposure for 7 min and 5 min exposure group, respectively. Corneal ulceration abated by day 7 post-exposure in the 7 min exposure group and by day 14 post-exposure in the 5 min exposure group. However, a slight increase in corneal ulceration was observed again on day 28 in the 7 min exposure group (Fig 1C).

Corneal NV was evaluated upon SM exposure in the eyes using a scoring method described previously [41]. This was based on the growth of the blood vessels in the cornea, calculated as percentage increase in the length of the blood vessels, taking the average NV from all four quadrants. Significant corneal NV was observed on day 7 post SM exposure in both the exposure groups (Fig 1D and S4 Dataset). Average corneal NV score on day 7 post-exposure was 0.58 for the 5 min exposure group and 0.56 for the 7 min exposure group; a gradual increase in NV score was observed thereafter at later time points. At the study end point i.e. on day 28 post SM exposure, the average corneal NV scores were 1.10 and 0.88 in the 5 min and 7 min exposure groups, respectively (Fig 1D).

### SM exposure causes increases in corneal thickness, epithelial degradation, and epithelial-stromal separation

**Fig 2:**
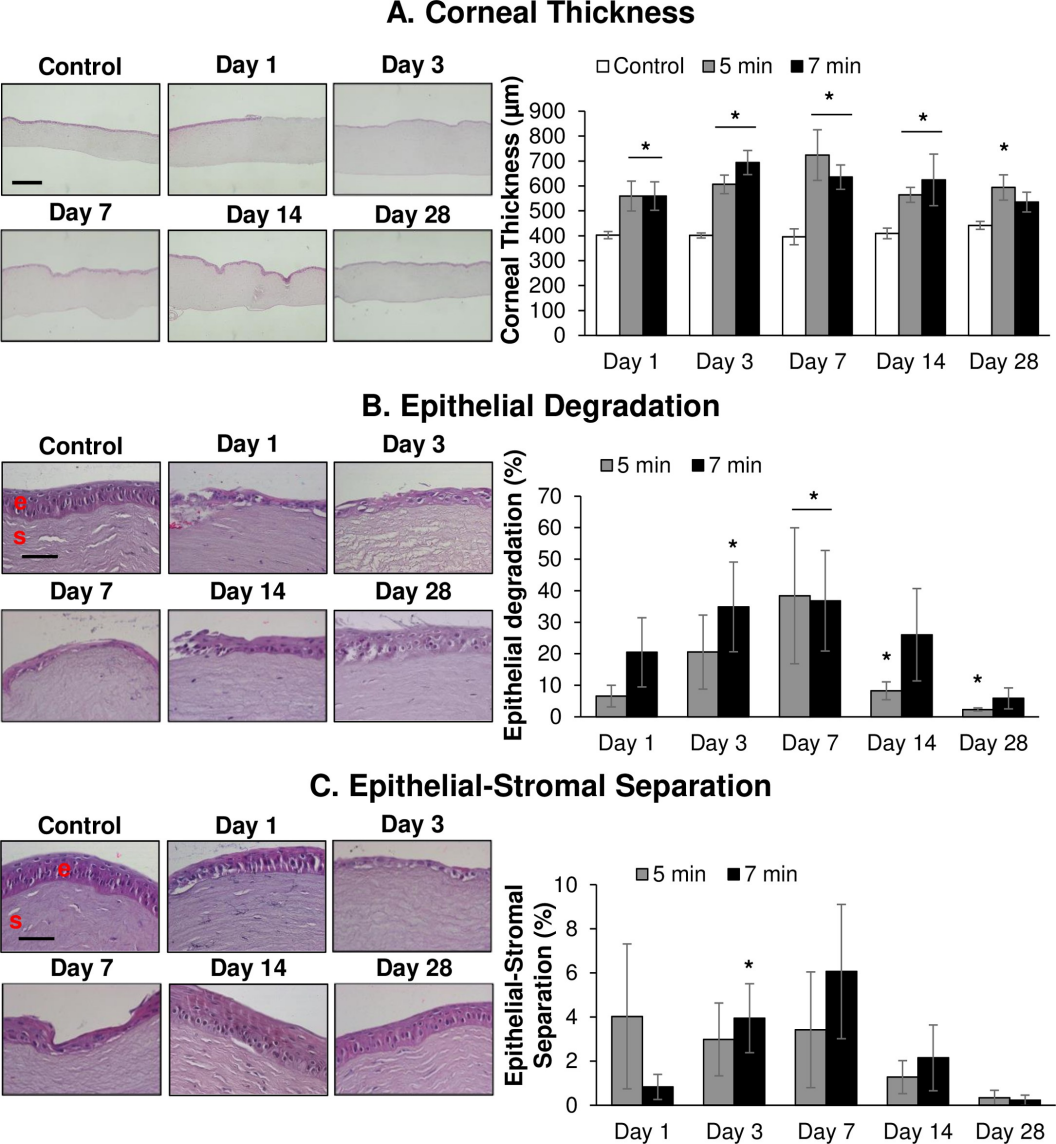
Ocular SM exposure causes increased corneal thickness and leads to epithelial degradation and epithelial-stromal separation. The right eyes of New Zealand white rabbits were exposed to SM (~0.4 mg/L) vapor either for 5 min (shorter duration) or 7 min (longer duration) and the clinical progression of ocular injury was observed starting from day 1 (6 h) up to 28 days post-exposure, as detailed in the materials and methods section. The left eye was designated as the control eye and was exposed only to the dilution air. Cornea was dissected post euthanasia at day 1, day 3, day 7, day 14, and day 28 post-exposure and histopathological evaluation. Corneal thickness and quantification (A), epithelial degradation and quantification (B), epithelial-stromal separation and quantification (C) in the H&E-stained control and SM-exposed sections was carried out as detailed under the materials and methods. Representative images are from the 7 min SM exposure. Data presented are mean±SEM (n = 3–5). *, p<0.05 compared to the control group; e, epithelium; s, stroma; red arrows, epithelial degradation/epithelial-stromal separation; size bar in representative images, 50 μm.

The histopathological alterations in the rabbit cornea following *in vivo* short and long durations of SM exposure are shown in Fig 2 in the H&E-stained corneal sections.

The increase in corneal thickness, as observed via pachymetry measurements performed *in vivo* rabbit eyes, was further confirmed by light microscopic evaluations of the H&E-stained corneal sections (Fig 2A and S5 Dataset). Initial increase in corneal thickness was observed as early as day 1 post-exposure, for both exposure groups and was significant at all the study time points (except on day 28 for 7 min SM exposure group). The maximal increase in corneal thickness for 7 min exposure group was observed on day 3 post-exposure (average corneal thickness was 694 μm in SM exposure group compared to 401 μm in control group). Whereas, in the 5 min exposure group the maximal increase was observed on day 7 post-exposure (average corneal thickness was 724 μm in SM exposure group compared to 396 μm in control group). The increase in corneal thickness started to resolve after the maximal increase at the later time points; although it was still elevated in both the exposure groups until the end point of the study on day 28 post-exposure, the difference between the untreated and SM-treated corneas was only significant for the 5 min exposure group (Fig 2A).

Epithelial degradation was observed since day 1 post SM exposure in both the exposure groups; however, the difference between the control and SM-exposed eyes was not significant on day 1 post-exposure (Fig 2B and S6 Dataset). An SM exposure duration-dependent effect was observed in terms of more degradation following 7 min exposure, which was significant on day 3 and maximal (~37% epithelial degradation) on day 7 post-exposure. The maximal and significant increase in the lower exposure group was observed on day 7 post-exposure (~38% epithelial degradation). The epithelial degradation decreased by day 14 post-exposure in both the exposure durations but was significant only for the 5 min exposure on day 14 and day 28 post-exposure. On day 28 post-exposure, ~2% and ~6% epithelial degradation was observed in the 5 min and 7 min post-exposure group, respectively (Fig 2B).

Separation of the epithelial-stromal layer was observed in both the exposure groups (Fig 2C and S7 Dataset) with significant separation only on day 3 post-exposure in the 7 min exposure group (~4% separation) and was maximal on day 7 (~6%) post-exposure. The epithelial stromal separation started to decrease by day 14 post-exposure and was almost negligible by day 28 post-exposure in both the exposure groups (Fig 2C).

### SM exposure causes keratocyte cell death, and increases in inflammatory cell density and number of blood vessels

**Fig 3:**
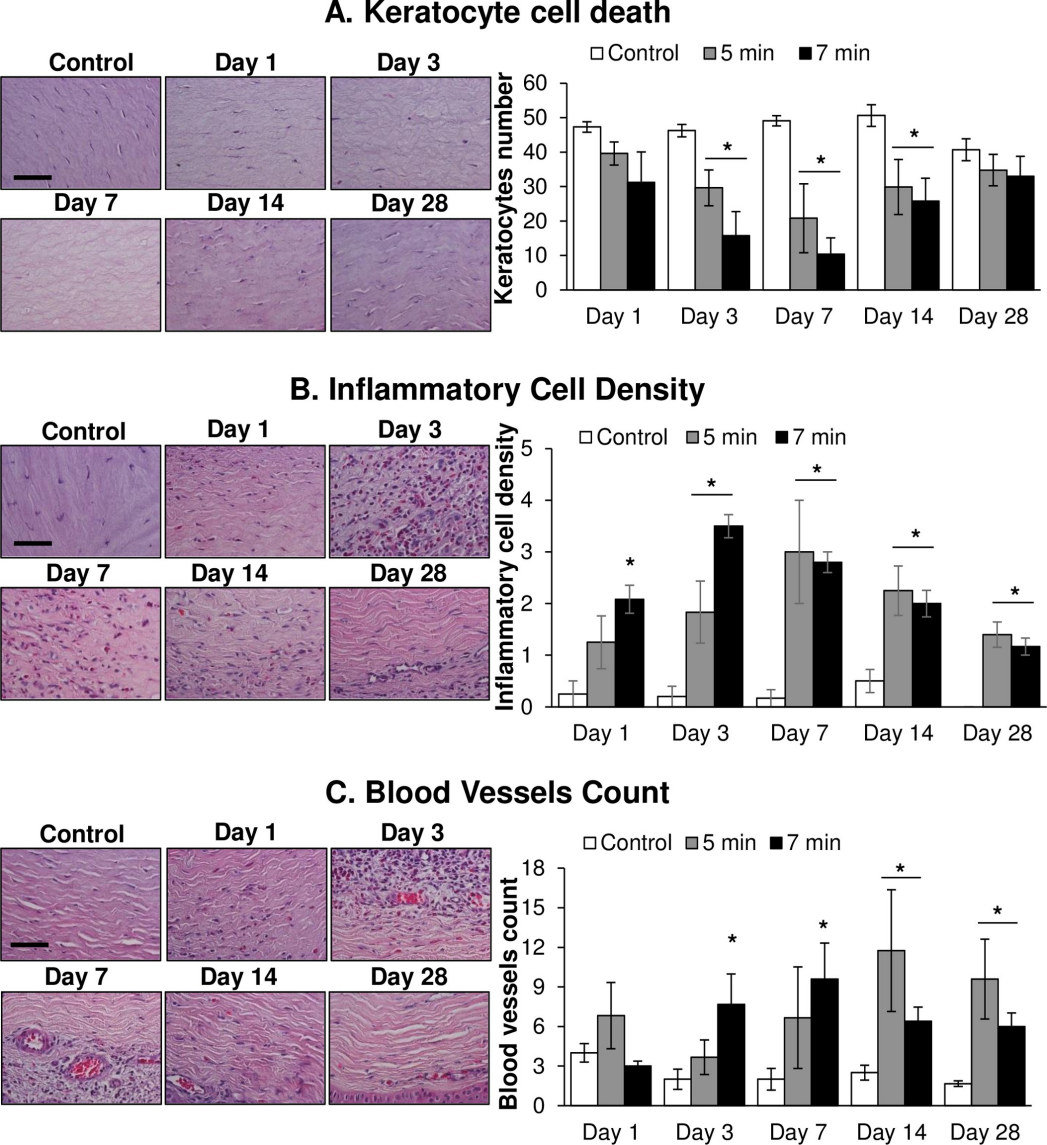
Ocular SM exposure causes keratocyte cell death, influx of inflammatory cells and increase in the number of blood vessels in the stroma. The right eyes of New Zealand white rabbits were exposed to SM (~0.4 mg/L) vapor either for 5 min (shorter duration) or 7 min (longer duration) and the clinical progression of ocular injury was observed starting from day 1 (6 h) up to 28 days post-exposure, as detailed in the materials and methods section. The left eye was designated as the control eye and was exposed only to the dilution air. Cornea was dissected post euthanasia at day 1, day 3, day 7, day 14, and day 28 post-exposure and histopathological evaluation was carried out. Number of keratocytes (A), inflammatory cells (B) and blood vessels (C) were quantified in H&E-stained corneal sections as detailed under the materials and methods. Data presented are mean±SEM (n = 3–5). *, p<0.05 compared to the control group; red arrows, keratocytes/inflammatory cells/blood vessels; size bar in representative images, 50 μm.

The alterations in keratocyte cells, inflammatory cells, and blood vessels in the rabbit cornea following *in vivo* short and long durations of SM exposure are shown in Fig 3 in H&E-stained sections.

SM exposure caused a decrease in keratocyte cell number starting from day 1 post-exposure (Fig 3A and S8 Dataset) in an exposure-dependent manner. A significant decrease in the keratocyte numbers was observed between the control and SM-exposed corneas on day 3 post SM exposure (average keratocyte number in 1 mm^2^ area of the stroma was ~46, 30, and 16 in the control, 5 min, and 7 min exposure groups, respectively). The drop in keratocyte number was maximal on day 7 post-exposure in both the 5 min and 7 min exposure groups (average keratocyte numbers in 1 mm^2^ area of the stroma were 49, 21, and 10 in control, 5 min, and 7 min exposure groups, respectively). The numbers of keratocytes began to increase on day 14 post-exposure, although they were still significantly lower than those in the control group (Fig 3A). On day 28 post-exposure, the keratocyte numbers were not significantly different between the control and SM exposure groups (Fig 3A).

SM exposure also led to an influx of inflammatory cells into the corneal stroma as observed on day 1 post SM exposure (Fig 3B and S9 Dataset), which was significant in the 7 min exposure group. An exposure-dependent effect was observed on day 1 and day 3. In the 7 min exposure group, number of inflammatory cells was maximal on day 3 post-exposure (average inflammatory cell density score was 3.5 in the SM exposure group compared to 0.2 in the control group) and started to decrease thereafter, although, it remained significantly higher in the SM exposure group compared to the control group at all the time points. In the 5 min exposure group, maximal influx of inflammatory cells was seen on day 7 post SM exposure (average inflammatory cell density score of 3.0 in the SM exposure group compared to 0.17 in the control group), which decreased on day 14 and day 28 post-exposure. However, the increase in inflammatory cell density was still significant at all the study time points in the 7 min exposure group (Fig 3B).

Ocular SM exposure resulted in an increase in the number of blood capillaries and vessels in the corneal stroma for both SM exposure durations (Fig 3C and S10 Dataset). The number of blood vessels increased significantly in the 7 min exposure group on day 7 post-exposure (average value 9.6 in the exposure group compared to 2.0 in the control group). Although there was a decrease in the number of blood vessels in the 7 min SM exposure group at the later time points of day 14 and day 28 post-exposure (6.4 and 6.0 in the exposure group compared to 2.5 and 1.7 in the control group, on day 14 and day 28, respectively), the number of blood vessels was still significantly higher in the 7 min SM-exposed group compared to the control group. In the 5 min exposure group, maximal increase in blood vessels was observed on day 14 post-exposure (average value 11.7 in the exposure group compared to 2.5 in the control group). On day 28 post-exposure, a decrease in the number of blood vessels was observed, although it was still significantly higher in the SM-exposed group than the control group (average value 9.6 in the exposure group compared to 1.7 in the control group; Fig 3C).

### SM exposure causes an increase in COX-2, VEGF, and MMP-9 expression

**Fig 4:**
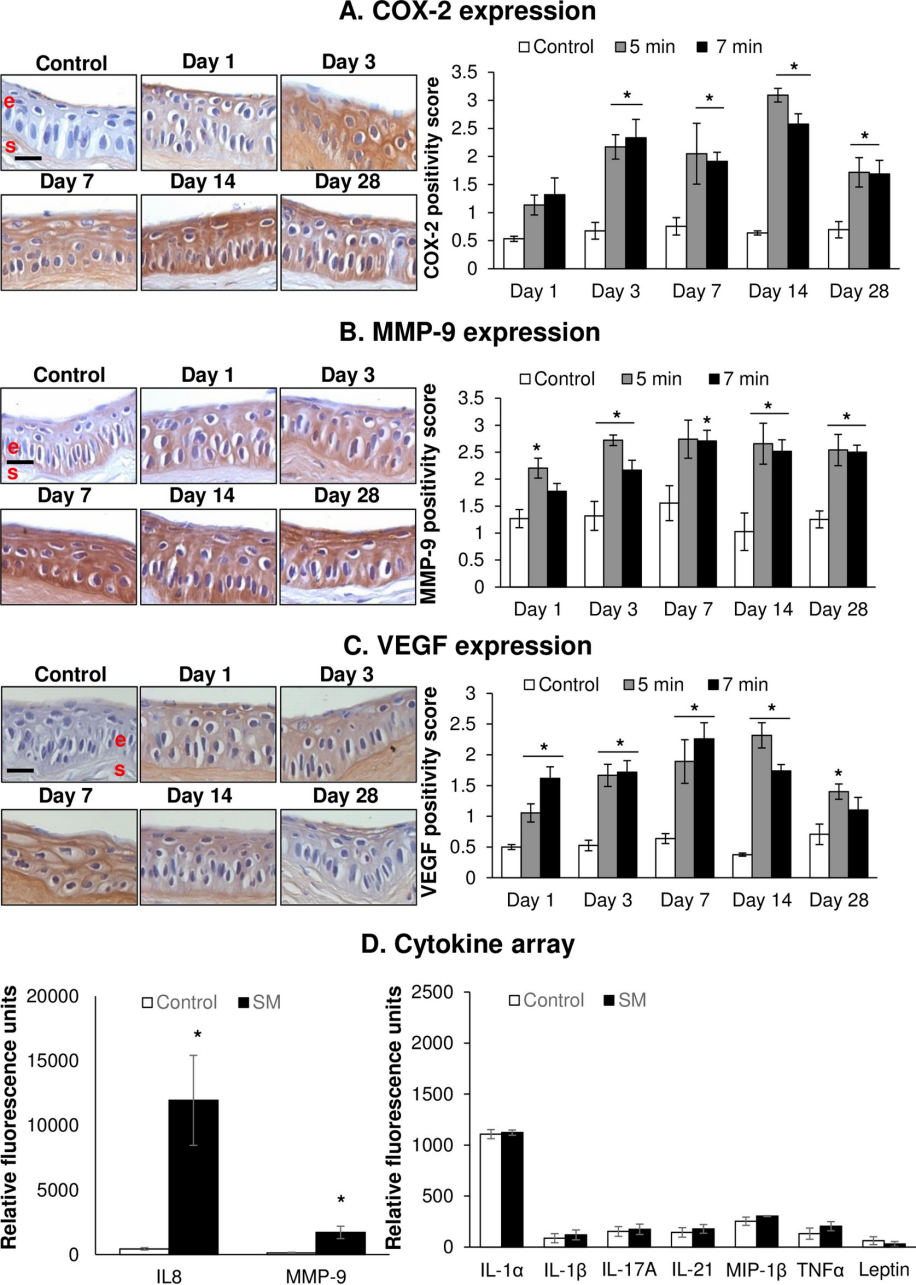
Ocular SM exposure causes increased expression of COX-2, VEGF, MMP-9, and IL-8. The right eyes of New Zealand white rabbits were exposed to SM (~0.4 mg/L) vapor either for 5 min (shorter duration) or 7 min (longer duration) and the clinical progression of ocular injury was observed starting from day 1 (6 h) up to 28 days post-exposure, as detailed in the materials and methods section. The left eye was designated as the control eye and was exposed only to the dilution air. Corneas were dissected post euthanasia at day 1, day 3, day 7, day 14, and day 28 post-exposure and fixed for IHC evaluation or frozen for cytokine array analysis. The corneal sections were IHC stained and COX-2 (A), MMP-9 (B), and VEGF (C) expression was quantified. Corneal lysates (7 min exposure group day 3 post-exposure) were prepared and subjected to cytokine array analysis (D) and relative fluorescence units for IL-8, MMP-9, IL-1α, IL-1β, IL-17A, IL-21, Macrophage Inflammatory Protein (MIP)-1β, TNF-α, and leptin were calculated. Only IL-8 and MMP-9 showed changes in expression levels upon SM-exposure; whereas, IL-1α, IL-1β, IL-17A, IL-21, MIP-1β, TNF-α, and leptin served as control cytokines, with similar expression in SM exposed and unexposed eyes. Data presented are mean±SEM (n = 3–5). *, p<0.05 compared to the control group; e, epithelium; s, stroma; size bar in representative images, 50 μm.

The alterations in COX-2, VEGF, and MMP-9 expression levels in the rabbit cornea following *in vivo* short and long durations of SM exposure are shown in Fig 4. SM exposure led to the increased expression of the inflammatory mediator COX-2 in the corneal epithelium, beginning on day 1 post SM exposure, which was significant on day 3 post-exposure for both the exposure durations (Fig 4A and S11 Dataset). The increase was maximal and significant on day 14 post-exposure for both the exposure durations as compared to the unexposed control (~4- and 5-fold increase for 5 min and 7 min exposures, respectively, in the SM exposure groups compared to the control group). The COX-2 expression decreased by day 28 post-exposure; however, it was still significantly higher in both the SM exposure groups compared to control group (Fig 4A).

Regarding MMP-9 expression, it increased on day 1 post SM exposure, which was significant in the 5 min exposure group (Fig 4B and S12 Dataset). At day 3 post-exposure, a significant increase in MMP-9 expression was noted in corneas exposed to both the exposure durations. Maximal increase in MMP-9 expression was observed on day 7 (~2-fold increase compared to control) post-exposure for both the exposure durations, which remained significantly elevated at comparable levels till day 28 post-exposure (Fig 4B). Cytokine array analysis further confirmed the MMP-9 expression increase observed upon SM exposure (Fig 4D and S14 Dataset). The 7 min SM exposure duration on day 3 post-exposure resulted in a ~13-fold increase in MMP-9 level compared to the control group (Fig 4D).

A significant increase in the expression of VEGF, a potent stimulator of angiogenesis, was observed beginning on day 1 post SM exposure for both the exposure durations and remained significantly elevated till day 14 post-exposure (Fig 4C and S13 Dataset). The increase in VEGF expression for the 5 min exposure group was significant at all time-points up to day 28 post SM exposure. The maximal increase in the VEGF expression as compared to control was observed on day 14 post-exposure for both the exposure durations (~6.0- and 4.6-fold increases, respectively, for 5 min and 7 min SM exposure groups compared to the control group).

Cytokine array analysis showed that SM exposure resulted in a 27-fold increase in the expression levels of inflammatory cytokine interleukin (IL)-8 compared to the control on day 3 post-exposure in the 7 min exposure group (Fig 4D). The concentrations of IL-1α, IL-1β, IL-17A, IL-21, Macrophage Inflammatory Protein (MIP)-1β, TNF-α, and leptin were similar between the SM exposed and control groups, which could be considered as control cytokines that did not show any change in concentration upon SM exposure (Fig 4D).

## Discussion

Even short durations of mild exposures to SM can cause substantial ocular injury, possibly because the eyes are the most sensitive organ to vesicant exposure [18, 25, 29]. The outer corneal layer is especially susceptible to the effects of SM toxicity, and exposures may lead to corneal abrasions, ulcerations, vesication, and perforations, the main consequences of acute corneal injury [18]. The signs of MGK and chronic effects of SM exposure on the cornea include corneal inflammation, ischemia, scarring, opacification, perforation, LSCD, and NV [20–25]. Effective and approved treatment modalities for the management of SM exposure-caused acute and chronic corneal injuries, especially in a mass-casualty situation are still not available. Our completed and ongoing studies, in addition to those done by other research groups, are focused on investigating the mechanism of corneal injury following mustard vesicant exposure. These studies could assist in deciphering pathways involved in generation of the SM-toxicity effects; furthermore, understanding and targeting key molecules in these pathways are essential for the development of effective treatment avenues and therapeutic interventions.

In the present study, we have assessed the corneal injury progression following two SM exposure-durations and evaluated the clinical, histopathological, and molecular endpoints of SM-induced corneal injury. The injury biomarkers, such as corneal opacity, corneal ulceration, corneal thickening, increases in keratocyte numbers, inflammatory cell density, and blood vessel counts, and expression levels of VEGF, COX-2, and MMP, established in our earlier reported studies in the NM-induced *ex vivo* and *in vivo* acute corneal injury models could be translated into the SM-induced *in vivo* corneal injury model in the present study [39, 40, 43]. As reported earlier, this study further corroborates that NM can serve as a useful surrogate to screen and identify effective therapies, which can be further tested in the SM-induced *in vivo* corneal injury model [12, 39, 44–47]. Our completed studies have shown beneficial effects of pleiotropic agents like dexamethasone and silibinin, which exhibit anti-inflammatory properties, in alleviating NM-induced corneal injury endpoints [40]. The parallel corneal injury endpoints established in the *in vivo* rabbit corneal injury model with SM in this study will be beneficial in further testing and optimizing these agents for their therapeutic efficacy against corneal injury caused by SM exposure.

Previous reports strongly indicate the alkylating effects of SM on ocular biomolecules, which may lead to inflammation, corneal degradation, and delayed LSCD and contribute to the pathogenesis of vesicant-induced ocular toxicity [16, 25, 48]. SM exposure is reported to induce histopathological changes including vesication, ulceration, keratocyte cell death, ocular inflammation, and NV [29, 49, 50]. In the present study, SM vapor exposure also resulted in corneal opacity, ulceration, NV, corneal epithelial degradation, keratocyte death, vesication, and an inflammatory response. In addition, SM also induced increases in the stromal blood vessel count, and the expressions of inflammatory markers COX-2 (important for prostaglandin synthesis and inflammatory and cytotoxic responses), MMP-9, VEGF, and IL-8. These injury lesions were comparable with the toxic corneal consequences from ocular SM exposure in humans [24, 51, 52].

Keratocytes and MMPs have been shown to play a crucial and complementary role in corneal wound healing mechanisms. Keratocytes are involved in the anabolism of collagen as well as secrete MMPs to facilitate their catabolism [53, 54]. These processes also regulate the transparent nature of the cornea by maintain a well-organized and dense packing of collagen fibrils in the cornea [55]. When there are disturbances in this balance, corneal opacity and delayed wound healing can be observed, amongst other effects. An association between keratocytes and epithelial cells of the cornea also exists to regulate the secretion of cytokines [56, 57]. Thus, an increased inflammatory response upon SM-exposure can lead to increased corneal ulceration and corneal opacity, mediated by the increased MMPs, as observed in the present study. The decrease in keratocyte cell number is also consistent with the findings of the previous study [35].

In clinical consequences, duration-dependent effects of SM exposure were observed for corneal thickness (pachymetry measurements), corneal opacity, and epithelial degradation (except day 7) in the present study. Corneal opacity was observed to be maximum on day 3 post SM exposure for both exposure durations; the opacity scores were elevated through day 28 post-exposure. Corneal ulceration peaked on days 1 and day 3 and was near completely resolved by day 7 and 14 for the 7 min and 5 min exposure duration, respectively. Our previous study on the NM-induced acute injury rabbit *in vivo* model, noted that both corneal opacity and ulceration showed a significant increase by day 3 post-exposure, and remained elevated till day 28 post-exposure [40]. The SM-induced epidermal degradation was maximal on day 3 post-exposure in both the exposure groups and could have contributed to the corneal ulceration and opacity. The alkylating property of SM, and its ability to induce oxidative stress and apoptotic cell death can contribute to the degradation of the epithelial layer leading to the disruption of permeability and barrier function of the cornea. Mechanistic studies in HCE cells have shown that NM-induced apoptotic cell death was associated with DNA damage conferred via p53 phosphorylation involving cleavage of caspase-3 and poly ADP ribose polymerase, suggesting their contribution in vesicant-induced corneal epithelial cell death [12]. The SM-induced keratocyte cell death was also found to be duration dependent. It significantly increased by day 3 post-exposure and was maximal on day 7 post-exposure, which could have resulted in corneal ulceration and perforation. Since keratinocytes are key molecules involved in maintaining and regenerating the cornea and regulate collagen synthesis, ROS related chemical injury from SM exposure could cause collagen degradation and affect the structural integrity of the stroma [45, 58]. Both SM exposure durations caused a significant increase in the corneal thickness starting on day 1 post-exposure, paralleling what we observed from our NM *in vivo* corneal injury model [40]. The observed SM-induced corneal thickness could be a consequence of the increased density of stromal inflammatory cells, starting on day 1 post SM exposure. In addition, the disruption of the epithelial barrier resulting in an altered hydration state of the cornea could also contribute to the corneal stromal thickness, as reported earlier [59].

Inflammation is reported to play a critical role in vesicating agent-induced corneal injury [18, 33, 40, 60]. As reported earlier in NM-induced *in vitro* and *in vivo* corneal injury models, the SM-induced increases in the MMP-9 corneal levels, in the current *in vivo* rabbit study, further establishes MMP-9 as a key mediator in mustard [46] vesicant-induced inflammation and epithelial-stromal separation [18, 39, 40, 43, 49]. There is evidence that the skin blistering, and epithelial-stromal separation observed in the cornea upon SM/NM exposure is because of the up-regulation of MMP-9 levels by the mustard vesicating agents [49, 61]. These studies have been carried out in SM-exposed war veterans, and SM and NM exposed animal models. Studies have shown that SM exposure-induced infiltration of neutrophils can elevate MMP-9, which can cleave the basement membrane and degrade the extracellular matrix leading to vesication, bullae formation, and cell death [46, 61–64].

The increases in the inflammatory mediator COX-2 levels observed upon both SM exposure durations at all the time points in this study paralleled those observed in our previous studies on HCE cells exposed to NM as well as the *ex vivo* and *in vivo* rabbit corneal injury models [12, 39, 40, 43]. The results from this study additionally endorse that vesicant-induced increase in COX-2, an inducible enzyme involved in prostaglandin biosynthesis, can enhance the influx of inflammatory cells in the skin dermis and corneal stroma [61, 65]. Exposure to both SM durations also resulted in a delayed NV response, which started after 7 days of SM exposure. Corneal NV has been extensively reported to be delayed consequence of vesicant ocular exposure that can lead to proliferation and migration of vascular endothelial cells into the stroma causing compromised visual acuity [12, 22, 27, 28, 32, 39, 43]. Proangiogenic mediators like VEGF can be secreted upon vesicant-induced inflammation by macrophages, corneal cells, and COX-2 levels can also enhance the expression of VEGF genes [66, 67]. Similar to NM exposure in *ex vivo* and *in vivo* rabbit corneal injury models, in the present study, SM ocular exposure also resulted in a significant increase in the expression of VEGF levels [40, 43, 49]. SM-induced increase in the IL-8 levels indicate that this chemokine can also contribute to the SM-related inflammation and angiogenesis [40, 68]. Increased levels of IL-8 have been previously reported in NM- and SM-induced corneal injury [22, 40, 42].

The observations from the present study demonstrate the effects of SM exposure in a temporal manner, encompassing histopathological and molecular changes, bridging structural changes with clinical outcomes via molecular mediators. Additionally, the relevant clinical, biological, and molecular markers of the corneal injury established in the *in vivo* rabbit model with SM in this study can be useful in testing and optimizing treatment agents.

## Supporting information

S1 Dataset Estimation of corneal thickness using pachymetry. (XLSX)

S2 Dataset Estimation of corneal opacity. (XLSX)

S3 Dataset Estimation of corneal ulceration. (XLSX)

S4 Dataset Estimation of corneal neovascularization. (XLSX)

S5 Dataset Estimation of corneal thickness. (XLSX)

S6 Dataset Estimation of epithelial degradation. (XLSX)

S7 Dataset Estimation of epithelial-corneal separation. (XLSX)

S8 Dataset Estimation of keratocyte count. (XLSX)

S9 Dataset Estimation of inflammatory cell count. (XLSX)

S10 Dataset Estimation of blood vessel count. (XLSX)

S11 Dataset Estimation of COX-2 expression. (XLSX)

S12 Dataset Estimation of MMP-9 expression. (XLSX)

S13 Dataset Estimation of VEGF expression. (XLSX)

S14 Dataset Cytokine array analysis. (XLSX)
